# Case Report: PTCH1 splice-site mutation and sonidegib treatment in Gorlin-Goltz syndrome: clinical insights from a family case study

**DOI:** 10.3389/fmed.2026.1778460

**Published:** 2026-02-24

**Authors:** Linli Liu, Heng Du, Neng Wang, Shuang Lv, Chunshui Yu, Lingli Deng

**Affiliations:** 1 Department of Dermatology, Suining Central Hospital, Suining, China; 2 Department of Gastroenterology, Suining Central Hospital, Suining, China

**Keywords:** basal cell carcinoma, Gorlin–Goltz syndrome, Hedgehog pathway inhibitor, minigene assay, PTCH1, sonidegib, splice-site variant

## Abstract

Gorlin-Goltz syndrome (nevoid basal cell carcinoma syndrome) is a rare autosomal dominant tumor-predisposition disorder characterized by multiple basal cell carcinomas (BCCs), odontogenic keratocysts of the jaws, and variable systemic manifestations. Although pathogenic variants in PTCH1 are a major genetic cause, transcript-level functional evidence for splice-site variants and real-world data on tolerability-oriented dosing of Hedgehog pathway inhibitors remain limited. We report a three-generation family in which a heterozygous canonical PTCH1 splice-donor variant (NM_000264.5:c.3449 + 1G > A) segregated with disease. A minigene splicing assay demonstrated exon 20 skipping, supporting a loss-of-function mechanism. Two affected relatives with symptomatic BCC burden received oral sonidegib for 6 months using different schedules (200 mg once daily vs. 200 mg every other day). Both patients showed clinical regression of target BCC lesions. Dysgeusia and alopecia occurred with daily dosing, whereas every-other-day dosing was well tolerated. This case highlights the value of transcript-level functional assays for interpreting PTCH1 splice-site variants and supports individualized, toxicity-guided sonidegib scheduling in selected patients with Gorlin-Goltz syndrome.

## 1 Introduction

Gorlin-Goltz syndrome (basal cell nevus syndrome; nevoid basal cell carcinoma syndrome) is a rare autosomal dominant disorder with high penetrance and variable expressivity, with an estimated prevalence ranging from 1/57,000 to 1/256,000 (1). Clinically, it is characterized by early-onset multiple BCCs and jaw odontogenic keratocysts, often accompanied by palmar/plantar pits, craniofacial features (e.g., macrocephaly), skeletal anomalies (e.g., bifid ribs), and intracranial calcifications such as falx cerebri calcification (1, 2). At the molecular level, Gorlin–Goltz syndrome is most commonly caused by pathogenic variants in PTCH1, a key negative regulator of Hedgehog (HH) signaling (3). PTCH1 encodes a transmembrane receptor that suppresses Smoothened (SMO) in the absence of ligand; germline loss-of-function in PTCH1 leads to constitutive HH pathway activation, providing a mechanistic basis for both developmental abnormalities and tumor susceptibility (4). Timely recognition is essential to enable risk-adapted surveillance and genetic counseling (5).

Constitutive HH pathway activation provides a clear therapeutic rationale for SMO inhibition. Oral SMO inhibitors, including vismodegib and sonidegib, can reduce BCC burden in patients with Gorlin–Goltz syndrome (6–8). However, continuous treatment is frequently limited by mechanism-based adverse effects, including dysgeusia, muscle cramps, alopecia, and weight loss (7, 8). In parallel, functional validation of PTCH1 splice-site variants together with real-world evidence supporting alternative dosing strategies remains scarce. Here, we describe a three-generation family with Gorlin–Goltz syndrome in which a PTCH1 canonical splice-donor variant was identified and functionally validated using a minigene assay, and we report clinical response and tolerability under two sonidegib dosing regimens.

## 2 Case description

**FIGURE 1:**
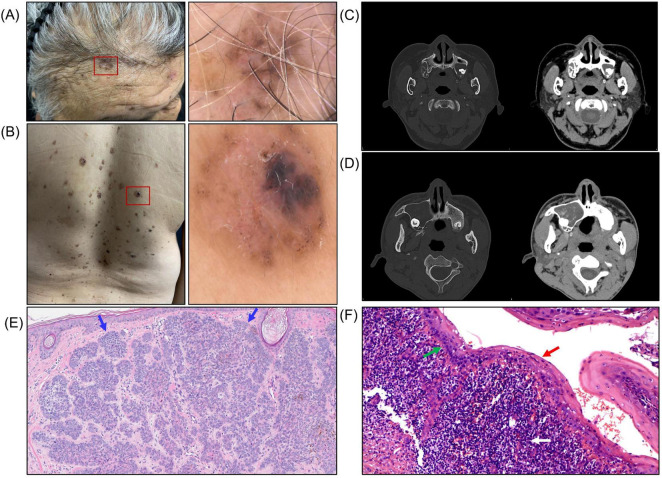
Clinical, dermoscopic, radiologic, and histopathologic findings in Patient 1 (proband) and Patient 2. **(A,B)** Clinical and corresponding dermoscopic images of multiple basal cell carcinomas (BCCs) on the scalp and trunk. **(C,D)** Craniofacial CT images demonstrating multiple odontogenic cysts. **(E)** Hematoxylin and eosin (H&E)–stained section of basal cell carcinoma showing dermal nests of basaloid cells with peripheral palisading (blue arrows). **(F)** H&E-stained section of an odontogenic cyst showing a stratified squamous epithelial lining (red arrow) with focal basal palisading (green arrow) and a dense inflammatory infiltrate in the cyst wall (white arrow).

Three members from the same family were evaluated in our dermatology department with a clinical history suggestive of an inherited tumor-predisposition syndrome. In addition to cutaneous findings, we conducted a targeted evaluation for characteristic features of Gorlin–Goltz syndrome, including palmar pits, craniofacial and skeletal abnormalities, as well as relevant radiologic assessments and jaw imaging. The proband (Patient 1), a 73-year-old woman, reported a 10-year history of recurrent dark brown-to-black cutaneous lesions accompanied by subcutaneous nodules. She had undergone multiple surgical excisions at outside hospitals, with histopathology confirming BCC on several occasions and epidermoid cysts in other excised nodules. On examination, multiple BCC lesions were present on the scalp and trunk, with dermoscopic documentation (Figures 1A, B). Craniofacial computed tomography (CT) revealed multiple odontogenic cysts (Figure 1C). Representative histopathology of an excised lesion showed basaloid nests with peripheral palisading on hematoxylin and eosin staining, consistent with BCC (Figure 1E). Histopathological examination of the jaw cyst demonstrated a stratified squamous epithelial lining with focal basal palisading and a dense inflammatory infiltrate in the cyst wall, consistent with an inflamed odontogenic cyst/odontogenic keratocyst (Figure 1F).

Her daughter (Patient 2), a 47-year-old woman, had developed progressive brown maculopapular lesions and multiple nodules over the preceding 5 years. She had previously undergone surgical excision of BCC and had been treated for an odontogenic cyst 1 year before presentation. Craniofacial CT similarly demonstrated multiple odontogenic cysts (Figure 1D). The proband’s grandson (Patient 3), an 18-year-old male, showed no clinical evidence of BCC at the time of evaluation but had a history of an odontogenic cyst treated 2 years earlier. The recurrence of BCC and odontogenic cysts across successive generations supported the clinical suspicion of Gorlin–Goltz syndrome.

**FIGURE 2:**
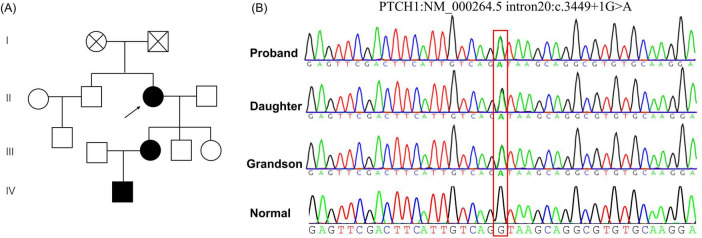
Pedigree and Sanger sequencing. **(A)** Pedigree consistent with autosomal dominant inheritance. **(B)** Representative Sanger sequencing electropherograms showing the heterozygous PTCH1 splice-donor variant (NM_000264.5:c.3449 + 1G > A) in affected family members and wild-type sequence in an unaffected control.

Given the familial aggregation and phenotypic variability, genetic testing was pursued for diagnostic confirmation and risk stratification. The pedigree was consistent with autosomal dominant inheritance (Figure 2A). Next-generation sequencing, followed by confirmatory Sanger sequencing, identified a heterozygous canonical splice-donor variant in PTCH1 (NM_000264.5:c.3449 + 1G > A) in affected family members; the variant was absent in an unaffected control (Figure 2B). Because the +1 position at the donor splice site is critical for normal pre-mRNA splicing, transcript-level functional validation was performed to define the splicing consequence of the variant.

**FIGURE 3:**
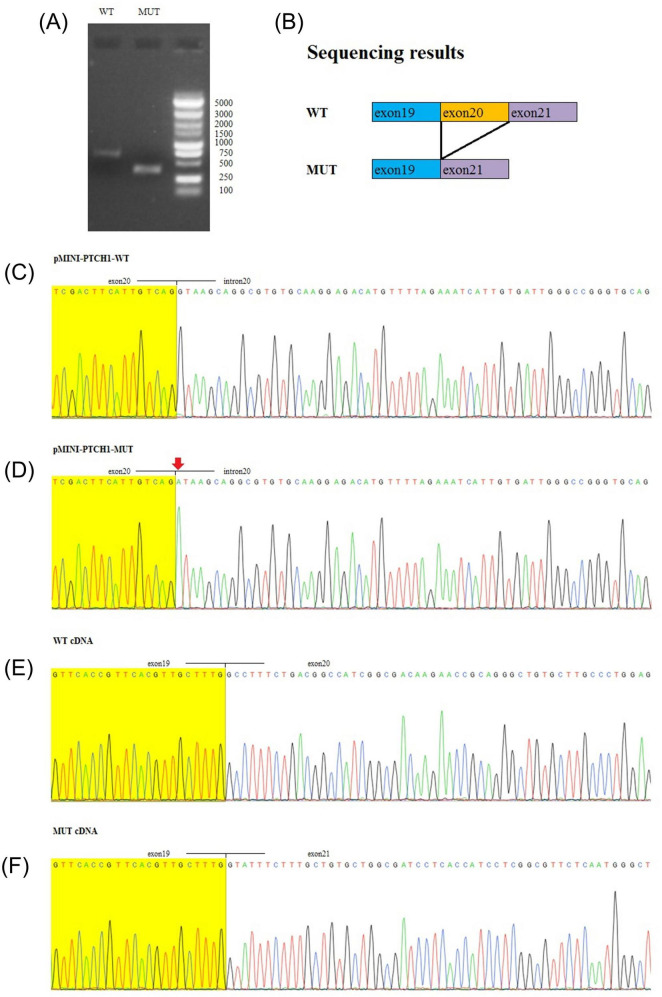
Minigene splicing assay demonstrating exon 20 skipping caused by PTCH1 c.3449 + 1G > A. **(A)** RT-PCR products from HEK293 cells transfected with wild-type (WT) or mutant (MUT) minigene constructs; the MUT construct yields a shorter amplicon consistent with exon 20 skipping. **(B)** Schematic representation of WT splicing and the mutant transcript lacking exon 20. **(C,D)** Sanger sequencing of the pMINI-PTCH1 plasmid inserts confirming the intron 20 donor-site variant. **(E,F)** Sanger sequencing of minigene-derived cDNA showing the canonical exon 19–20 junction in WT and an aberrant exon 19–21 junction in MUT. WT, wild type; MUT, mutant.

To assess the transcript-level consequence of PTCH1 c.3449 + 1G > A, a minigene splicing assay was performed. Wild-type and mutant minigene constructs were transiently expressed in HEK293 cells, and transcripts were analyzed by RT-PCR. Compared with the wild-type construct, the mutant construct generated a shorter RT-PCR product (Figure 3A), consistent with exon 20 skipping as schematically illustrated (Figure 3B). Sanger sequencing confirmed the intron 20 donor-site substitution in the mutant plasmid insert (Figures 3C, D). Sequencing of RT-PCR products demonstrated the expected exon 19–20 junction in wild type, whereas mutant transcripts showed an aberrant exon 19–21 junction, confirming complete skipping of exon 20 (Figures 3E, F).

**FIGURE 4:**
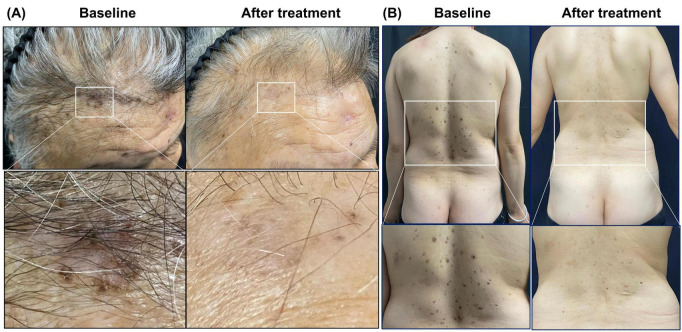
Clinical response to sonidegib in Patients 1 and 2. **(A)** Patient 1: scalp and forehead before and after 6 months of sonidegib treatment (200 mg once daily), with magnified views showing flattening and fading of target lesions. **(B)** Patient 2: posterior trunk before and after 6 months of sonidegib treatment (200 mg every other day), with magnified views highlighting regression of representative lesions.

Given the clinical burden of recurrent BCCs in Patients 1 and 2 despite repeated surgical interventions, systemic HH pathway inhibition was considered. Sonidegib was initiated with individualized dosing schedules. Baseline and periodic creatine kinase and renal function monitoring was performed, and pregnancy prevention counseling/testing was provided as appropriate. Patient 1 received sonidegib (ODOMZO) 200 mg once daily and Patient 2 received 200 mg every other day, both for a total duration of 6 months. During treatment, both patients exhibited clinical improvement of target lesions, including flattening, softening, and regression (Figures 4A, B). Patient 1 showed a more pronounced response on the scalp and forehead but developed dysgeusia and severe hair loss during therapy. These adverse events were managed conservatively with patient counseling, dietary advice (including the intake of bland, naturally flavored foods and maintenance of oral hygiene), and close follow-up; for alopecia, supportive measures such as wig or hat use were recommended, and the patient elected to continue therapy. Patient 2 demonstrated regression of lesions on the posterior trunk without notable adverse events. Patient 3 remains under surveillance given his history of odontogenic cysts and the familial genetic background.

## 3 Discussion

Hedgehog signaling has crucial roles in embryonic tissue patterning, adult tissue homeostasis, and carcinogenesis (9). Canonical HH signaling is initiated when HH ligands bind to PTCH1, thereby relieving PTCH1-mediated suppression of SMO and triggering downstream activation of GLI transcription factors. In Gorlin–Goltz syndrome, germline loss-of-function variants in PTCH1 result in constitutive HH pathway activation and confer a marked lifelong predisposition to developing multiple BCCs (10, 11). This well-defined molecular pathogenesis supports targeted SMO inhibition to reduce tumor burden and cumulative morbidity from repeated local treatments. Sonidegib, an orally available SMO antagonist, has demonstrated clinically meaningful activity in advanced BCC and, in Gorlin–Goltz syndrome, has been associated with reductions in BCC counts and/or clearance of target lesions (6, 12).

Our report provides transcript-level functional evidence for PTCH1 c.3449 + 1G > A by demonstrating exon 20 skipping in a minigene assay, directly supporting a pathogenic splice defect. The PTCH1 c.3449 + 1G > A variant affects a canonical splice donor site and is expected to disrupt mRNA splicing. In ClinVar, this variant is classified as pathogenic (RCV000195968.6) and is reported as not present in population databases (ExAC no frequency), supporting that it is extremely rare. While syndrome-level penetrance for PTCH1-related Gorlin–Goltz syndrome is high, expressivity is variable and not all typical manifestations are observed or documented in every affected individual (2). In the present family, the variant segregated with jaw cysts across generations and with BCC development in two affected individuals, supporting its clinical relevance in this pedigree. Minigene-based splicing assays are particularly valuable when patient-derived RNA is unavailable or when relevant isoforms are not expressed in accessible tissues, and RNA-level evidence is increasingly incorporated into clinical variant interpretation workflows (13–15).

Clinically, both continuous daily and intermittent sonidegib schedules led to regression of BCC lesions in our patients. Daily dosing produced a more pronounced response but was limited by dysgeusia and alopecia, whereas an every-other-day regimen was better tolerated with maintained benefit. This trade-off aligns with broader experience that class-related toxicities frequently drive dose interruptions or schedule adjustments in patients receiving HH pathway inhibitors, and that such modifications can help maintain adherence (16). Consistent with this, a national real-life cohort reported no statistically significant difference in clinical improvement between daily and 48-h sonidegib schedules (17). Gorlin-focused series have also described that schedule adjustments can improve tolerability without clear loss of efficacy, supporting individualized, toxicity-guided dosing strategies (18, 19).

Smoothened inhibitors share a characteristic adverse event spectrum related to on-target HH pathway inhibition. Common adverse events include muscle cramps, dysgeusia, alopecia, fatigue, nausea, decreased appetite, and weight loss (7, 8, 16). For sonidegib, additional monitoring for creatine kinase elevation and musculoskeletal toxicity is recommended (12). Management is mainly supportive, emphasizing patient education, symptom monitoring, nutritional/oral-hygiene guidance, and, when needed, dose interruption or intermittent dosing to improve tolerability (16). For reference, the SMO inhibitors employed in GGS/NBCCS management and their key pharmacokinetic and safety considerations are summarized in Table 1.

**TABLE 1:** Overview of Smoothened (SMO) inhibitors employed for Gorlin-Goltz syndrome (GGS)/nevoid basal cell carcinoma syndrome (NBCCS) management.

Agent	Indication	Mechanism	Key PK features	Rationale in GGS/NBCCS	Key safety notes/evidence
Vismodegib (GDC-0449; Erivedge)	Approved for advanced BCC (metastatic or locally advanced not amenable to surgery/radiation); used in NBCCS/GGS to reduce BCC burden	Oral Smoothened (SMO) antagonist; inhibits Hedgehog (HH) signaling downstream of *PTCH1*	Non-linear pharmacokinetics with high plasma protein binding (>97%) and moderate distribution (Vd ∼16–27 L); supports effective once-daily dosing in clinical studies (7, 8, 16).	Targets constitutive HH activation in PTCH1-driven disease, reducing new and existing BCCs and cumulative need for repeated local procedures	Class AEs: dysgeusia, muscle cramps, alopecia, weight loss; embryo-fetal toxicity. NBCCS trial evidence: (7, 8).
Sonidegib (LDE225; Odomzo)	Approved for locally advanced BCC; evaluated in NBCCS/GGS cohorts and supports toxicity-guided/intermittent schedules	Oral SMO antagonist; HH pathway inhibition	Long terminal half-life (∼28 days) with extensive distribution (Vd > 9000 L) and high protein binding (>99%); primarily CYP3A metabolism; exposure increases with high-fat meals (12, 16).	Alternative systemic SMO inhibitor for long-term HH suppression in NBCCS/GGS; dosing can be individualized to balance response and tolerability	Class AEs; monitor creatine kinase (CK) and musculoskeletal toxicity. NBCCS trial evidence: (12).
Topical sonidegib (LDE225) 0.75% cream (investigational)	Investigational topical SMO inhibitor studied in NBCCS/GGS-related BCCs (lesion/field-directed)	Topical SMO antagonist; local HH pathway inhibition in BCC tissue	Designed for local skin exposure with minimal systemic levels	Potential to treat multiple superficial/field lesions while minimizing systemic class toxicities	Mainly local reactions; systemic AEs uncommon in topical study. Evidence: (23).
Patidegib topical gel (investigational)	Investigational topical HH/SMO inhibitor in Gorlin–Goltz syndrome (NBCCS/GGS), including facial field therapy approaches	Topical SMO antagonist (cyclopamine-derived)	Local delivery with low systemic exposure	Aims to reduce/prevent new facial BCCs with improved tolerability versus systemic therapy	Generally mild local AEs; minimal systemic class toxicity reported. Evidence: (22).
Saridegib (IPI-926) (investigational)	Investigational oral SMO inhibitor studied in advanced BCC; limited human evidence in Gorlin–Goltz syndrome within trial/case context	Oral SMO antagonist	Clinical PK per early-phase oncology development; limited GGS-specific PK data available	Represents additional SMO inhibitor that has been employed in HH-driven BCC contexts; GGS-specific use remains limited/experimental	Class AEs expected; availability limited. Evidence/case context: (24).

BCC, basal cell carcinoma; GGS, Gorlin–Goltz syndrome; NBCCS, nevoid basal cell carcinoma syndrome; HH, Hedgehog; PK, pharmacokinetics; SMO, Smoothened.

However, despite these benefits, clinical efficacy may be limited by primary or acquired resistance, which can involve SMO mutations reducing drug binding, downstream HH activation (e.g., GLI2), or bypass signaling such as PI3K (20). A recent multi-omics/spatial transcriptomics study in a long-term SMOi–treated Gorlin patient suggested that resistance may also arise via a basal-to-squamous transition (BST) (21). In parallel, topical HH pathway inhibition may offer lesion- or field-directed control with reduced systemic toxicity; a phase II trial of patidegib topical gel in Gorlin–Goltz syndrome reported suppression of HH signaling with minimal systemic adverse effects (22). Nevertheless, topical therapy is unlikely to suffice for extensive or deeply invasive disease, and systemic therapy was therefore selected in our setting given the overall lesion burden and prior repeated surgery.

In conclusion, we report a three-generation family with Gorlin-Goltz syndrome in whom a heterozygous PTCH1 canonical splice-donor variant (NM_000264.5:c.3449 + 1G > A) segregated with disease. A minigene assay confirmed exon 20 skipping, supporting a pathogenic splicing defect. Oral sonidegib reduced BCC burden over 6 months in two affected relatives; daily dosing was limited by dysgeusia and alopecia, whereas every-other-day dosing was better tolerated with maintained benefit. These findings support RNA-level functional testing for PTCH1 splice-site variants and suggest toxicity-guided sonidegib scheduling as a practical component of individualized long-term management in Gorlin–Goltz syndrome.

## Data Availability

The original contributions presented in this study are included in this article/supplementary material, further inquiries can be directed to the corresponding authors.
